# 6‐Dihydroparadol, a Ginger Constituent, Enhances Cholesterol Efflux from THP‐1‐Derived Macrophages

**DOI:** 10.1002/mnfr.201800011

**Published:** 2018-06-25

**Authors:** Dongdong Wang, Verena Hiebl, Angela Ladurner, Simone L. Latkolik, Franz Bucar, Elke H. Heiß, Verena M. Dirsch, Atanas G. Atanasov

**Affiliations:** ^1^ Department of Pharmacognosy University of Vienna Althanstrasse 14 1090 Vienna Austria; ^2^ Institute of Genetics and Animal Breeding of the Polish Academy of Sciences ul. Postepu 36A 05–552 Jastrzebiec Poland; ^3^ Institute of Clinical Chemistry University Hospital Zurich University of Zurich Wagistrasse 14 8952 Schlieren Switzerland; ^4^ Department of Pharmacognosy, Institute of Pharmaceutical Sciences University of Graz 8010 Graz Austria

**Keywords:** atherosclerosis, ATP‐binding cassette transporter A1, ATP‐binding cassette transporter G1, cardiovascular diseases, cholesterol efflux

## Abstract

**Scope:**

Ginger is reported to be used for the prevention and treatment of cardiovascular diseases (CVD). Cholesterol efflux from macrophage foam cells is an important process in reverse cholesterol transport, whose increase may help to prevent or treat CVD. In this study, we investigated the effects of 6‐dihydroparadol from ginger on macrophage cholesterol efflux.

**Methods and results:**

We show that 6‐dihydroparadol concentration‐dependently enhances both apolipoprotein A1‐ and human plasma–mediated cholesterol efflux from cholesterol‐loaded THP‐1‐derived macrophages using macrophage cholesterol efflux assay. 6‐Dihydroparadol increases protein levels of both ATP‐binding cassette transporters A1 and G1 (ATP‐binding cassette transporter A1 [ABCA1] and ATP‐binding cassette transporter G1 [ABCG1]) according to Western blot analysis. The ABCA1 inhibitor probucol completely abolishes 6‐dihydroparadol‐enhanced cholesterol efflux. Furthermore, increased ABCA1 protein levels in the presence of 6‐dihydroparadol were associated with both increased ABCA1 mRNA levels and increased ABCA1 protein stability. Enhanced ABCG1 protein levels were only associated with increased protein stability. Increased ABCA1 protein stability appeared to be the result of a reduced proteasomal degradation of the transporter in the presence of 6‐dihydroparadol.

**Conclusion:**

We identified 6‐dihydroparadol from ginger as a novel promoter of cholesterol efflux from macrophages that increases both ABCA1 and ABCG1 protein abundance. This newly identified bioactivity might contribute to the antiatherogenic effects of ginger.

## 1 Introduction

Ginger, *Zingiber officinale* Roscoe (Zingiberaceae), is widely used around the world as a spice and flavoring agent.^1^ Rhizomes of ginger, in addition to their culinary application, have been used since ancient times for the therapy of a variety of conditions, including colds, fevers, nausea and digestive problems, and as an appetite stimulant.^1^ In recent decades, ginger has been studied extensively in animal and in vitro models, leading to observations for its activity as an antioxidant,^2^ anti‐inflammatory,^2^ antimicrobial,^3^ anticarcinogenic,^4^ and analgesic agent.^4^ Epidemiological and clinical studies from recent years have built a consensus that ginger and its major pungent constituents (i.e., gingerols, shogaols, and paradols) exert beneficial effects against metabolic disorders including obesity, nonalcoholic fatty liver disease, and diabetes.^5^

Several studies also suggested that consumption of ginger and its major bioactive constituents are associated with decreased risk of cardiovascular diseases (CVD), such as hypertension and atherosclerosis.^5^, ^6^ Ginger was reported to be used for the prevention of hypertension, which is supported by studies showing the inhibition of angiotensin‐1‐converting enzyme by ginger^7^ as well as the inhibition of angiotensin II type 1 receptor (AT_1_) activation by 6‐gingerol.^8^ Ginger and its major bioactive constituents also led to a significant reduction in platelet aggregation, which plays a central role in hemostasis and thrombosis.^9^ It is worth noting that ginger has been documented to exhibit positive effects on atherosclerosis, which is the main cause for CVD.^10^ It was found that the antiatherogenic effect of ginger was associated with a significant reduction in plasma LDL, triglycerides and cholesterol levels, the inhibition of LDL oxidation, as well as an increase in HDL levels.^11^

Although previous studies have addressed the molecular mechanisms of the antiatherogenic effect of ginger or its bioactive constituents, it remains to be established whether ginger components influence cholesterol efflux from macrophage foam cells, which is a promising strategy for the prevention and treatment of atherosclerosis.^12^ Formation and accumulation of macrophage foam cells containing excessive oxidized LDL‐derived cholesterol in the subendothelial area of the arterial wall is a hallmark of atherosclerosis.^13^ Recent clinical reports indicated that increased macrophage cholesterol efflux is significantly and inversely associated with CVD, suggesting that the macrophage cholesterol efflux capacity may be a novel predictive biomarker for the incidence of cardiovascular events.^13^ Cholesterol efflux from macrophage foam cells mainly involves the active transport of cholesterol to the acceptors apolipoprotein A1 (apo A1) and HDL by ATP‐binding cassette transporter A1 (ABCA1) and ATP‐binding cassette transporter G1 (ABCG1)/scavenger receptor class B type I (SR‐BI), respectively.^14^ *ABCA1* and *ABCG1* gene expression is predominantly upregulated by the two isoforms of the nuclear receptor liver X receptor (LXRα/β), which form a permissive heterodimer with the retinoid X receptor (RXR) in order to act as transcription factors. The expression of ABCA1, ABCG1, SR‐BI, and LXR can also be induced by activation of the peroxisome proliferator activated receptor‐γ (PPARγ).^15^

In this study, we investigated the influence of four pungent compounds present in ginger (6‐gingerol, 6‐shogaol, 6‐paradol, and 6‐dihydroparadol)^16^ on macrophage cholesterol efflux and pursued the cellular mode of action of the identified active compound, 6‐dihydroparadol.

## 2 Experimental Section

### 2.1 Materials

**Figure 1:**
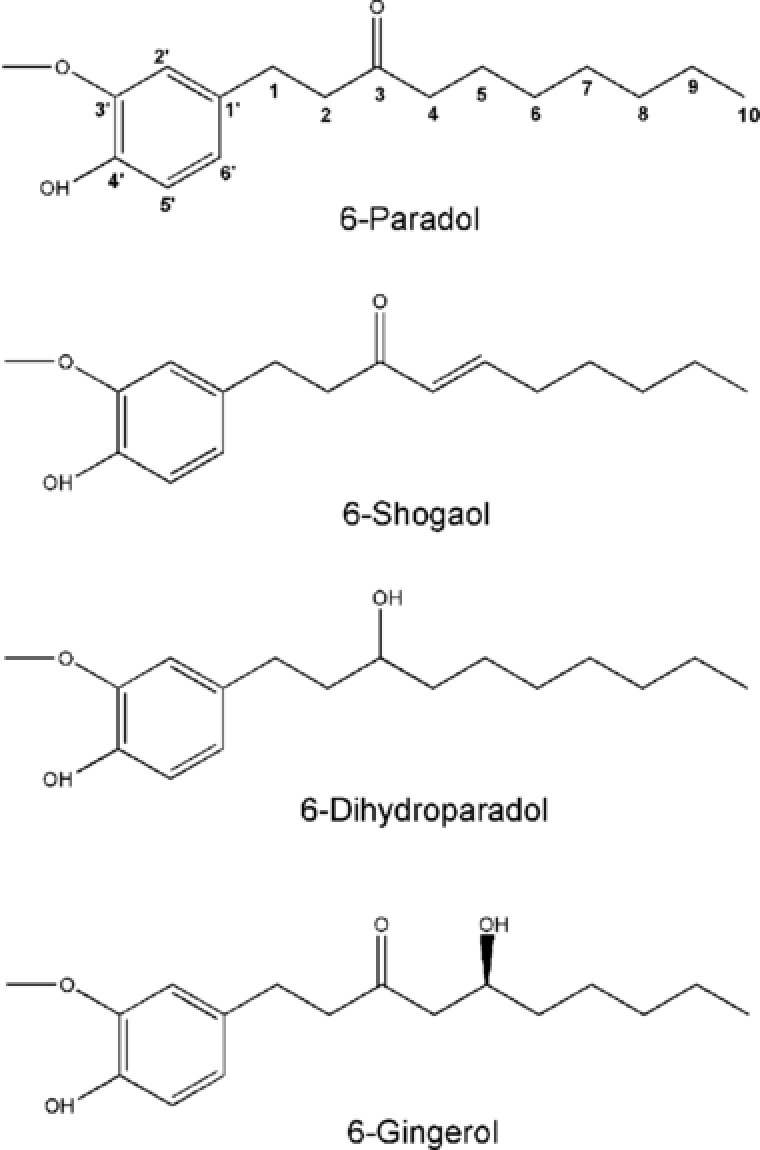
Chemical structures of the pungent components present in ginger, *Zingiber officinale* Roscoe, studied in this work.

6‐Paradol and 6‐dihydroparadol (*rac*‐6‐dihydroparadol) were isolated as described,^17^ while 6‐gingerol and 6‐shogaol were obtained commercially from Sigma‐Aldrich (Vienna, Austria). The chemical structures of the four compounds are shown in **Figure** 1. Resazurin sodium salt (catalog no. R7017), digitonin (catalog no. D141), phorbol 12‐myristate 13‐acetate (PMA; catalog no. P1585), apo A1 (catalog no. SRP4693), TO901317 (catalog no. T2320), GW3965 hydrochloride (catalog no. G6295), pioglitazone (catalog no. E6910), bexarotene (catalog no. SML0282), cycloheximide (CHX; catalog no. C7698), lactacystin (catalog no. L6785), calpeptin (catalog no. C8999), chloroquine diphosphate salt (catalog no. C6628), probucol (catalog no. P9672), and water‐soluble cholesterol (catalog no. C4951) were also purchased from Sigma‐Aldrich (Vienna, Austria). Fatty acid‐free BSA was obtained from Carl Roth (Karlsruhe, Germany). [^3^H]‐cholesterol (catalog no. NET139001MC) was provided by PerkinElmer (Traiskirchen, Austria). RPMI‐1640 and DMEM medium were purchased from Lonza (Basel, Switzerland). Fetal bovine serum (FBS) and trypsin were obtained from Gibco via Invitrogen (Lofer, Austria). Human plasma was obtained from young, healthy volunteers. The human LXRα, human LXRβ, and human RXRα expression plasmids (each pcDNA3.1+) were obtained from Missouri S&T cDNA Resource Center (Rolla, MO). The human PPARγ expression plasmid (pSG5‐PL‐hPPAR‐γ1) was a kind gift from Prof. Beatrice Desvergne and Prof. Walter Wahli (Center for Integrative Genomics, University of Lausanne, Switzerland).^18^ The luciferase reporter plasmids ABCA1‐Luc (pGL4.14), RXRE‐Luc (pTL‐Luc), and PPRE‐Luc (tk‐PPREx3‐luc) were kindly provided by Dr. Irena Ignatova (University of Virginia Health System, Charlottesville, VA),^19^ obtained from Panomics Srl (Milan, Italy), and kindly provided by Prof. Ronald M. Evans (Howard Hughes Medical Institute, La Jolla, CA),^20^ respectively. Enhanced green fluorescence protein expression plasmid (pEGFP‐N1) was purchased from Clontech Laboratories (Mountain View, CA). Reporter lysis 5 × buffer (catalog no. E3971), which was used with the luciferase assay system, was purchased from Promega (Madison, WI). The tested compounds were dissolved in DMSO at 30 mm, and stored at −20 °C. The final DMSO concentration for each condition in all experiments is 0.1%.

Primary antibodies against ABCA1 (catalog no. NB400‐105), ABCG1 (catalog no. NB400‐132), and SR‐BI (catalog no. NB400‐104) were obtained from Novus Biologicals (Vienna, Austria). The anti‐actin antibody (catalog no. 8691002) was acquired from MP Biologicals (Illkirch, France). Goat anti‐mouse secondary antibody, HRP conjugate (catalog no. 12–349) was purchased from Millipore (Vienna, Austria), and anti‐rabbit IgG, HRP‐linked secondary antibody (catalog no. 7074S) was obtained from Cell Signaling via New England Biolabs (Frankfurt am Main, Germany). The peqGOLD Total RNA Kit (catalog no. 732–2868) was purchased from PeqLab (Linz, Austria), and the High Capacity cDNA Reverse Transcription Kit (catalog no. 4368813) was from Applied Biosystems (Vienna, Austria). The LightCycler 480 SYBR Green I Master (catalog no. 04707516001) was from Roche (Mannheim, Germany). ABCA1 (Hs_ABCA1_1_SG QuantiTect Primer Assay; catalog no. QT00064869), ABCG1 (Hs_ABCG1_1_SG QuantiTect Primer Assay; catalog no. QT00021035), and human 18S (Hs_RRN18S_1_SG QuantiTect Primer Assay; catalog no. QT00199367) oligonucleotide primers were purchased from Qiagen (Vienna, Austria).

### 2.2 Cell Culture

THP‐1 cells and HEK293 cells (both obtained from ATCC, Manassas, VA) were maintained, respectively, in RPMI‐1640 medium and DMEM medium (without phenol red supplemented with 4.5 g L^−1^ glucose) supplemented with 10% heat‐inactivated FBS, 100 U mL^−1^ benzylpenicillin and 100 μg mL^−1^ streptomycin, and 2 mm l‐glutamine in a humidified atmosphere at 37 °C and 5% CO_2_. The THP‐1 cells and HEK293 cells were cultured in T175 flasks, and passaged every 2 days.

### 2.3 Cholesterol Efflux Assay

The cholesterol efflux assay was performed in line with previously published studies.^21^ THP‐1 cells were seeded at a density of 0.2 × 10^6^ mL^−1^ in a volume of 1 mL per well in 24‐well plates and differentiated into macrophages for 72 h with 200 nm PMA in RPMI‐1640 medium containing 10% FBS. After being washed twice with PBS, macrophages were labeled by incubation in RPMI‐1640 medium (0.5 mL per well) supplemented with 2.5% FBS, [^3^H]‐cholesterol (0.3–0.5 μCi mL^−1^) and cholesterol (20 μg mL^−1^) for 24 h.^21^

To evaluate the effect of bioactive components present in ginger on macrophage cholesterol efflux, labeled cells were washed with PBS and then treated with the different compounds (6‐paradol [30 μm], 6‐shogaol [10 μm], 6‐dihydroparadol [30 μm], 6‐gingerol [30 μm], TO901317 [5 μm, positive control]) or solvent vehicle (0.1% DMSO) for another 24 h. Cells were washed again with PBS and then incubated with fresh serum‐free medium containing human plasma (1%, v/v) for 6 h to induce macrophage cholesterol efflux. For the concentration‐dependent experiments, labeled cells were washed with PBS and treated with different concentrations of 6‐dihydroparadol (0, 0.3, 1, 3, 10, or 30 μm) or TO901317 (1 or 5 μm, positive control) for 24 h. Cells were washed again with PBS and then incubated with fresh serum‐free medium containing human plasma (1%, v/v) or apo A1 (10 μg mL^−1^) for 6 h to induce macrophage cholesterol efflux. In order to estimate the contribution of the ABCA1 transporter to the 6‐dihydroparadol‐increased cholesterol efflux, labeled cells were washed with PBS and treated with 6‐dihydroparadol (15 μm), probucol (20 μm), or co‐treated with 6‐dihydroparadol and probucol (inhibiting ABCA1 membrane localization and ABCA1‐mediated transport^22^) for 24 h. Cells were washed again with PBS and then incubated with fresh serum‐free medium containing human plasma (1%, v/v) and corresponding compounds for another 6 h to induce macrophage cholesterol efflux. Effluxed (medium supernatant) and intracellular (cell lysate) [^3^H]‐cholesterol were counted by liquid scintillation. Cholesterol efflux (percentage of total cholesterol) was determined by the ratio of radio‐labeled cholesterol in the medium to that of both medium and cells.^21^ The specific efflux is calculated as the difference between the efflux in the presence and absence of the acceptor (blank): Specific efflux (%) = Cholesterol efflux (%) − Blank efflux (%).^21^

### 2.4 Resazurin Conversion Assay

Influence of 6‐dihydroparadol on cell viability was assessed by the resazurin conversion assay.^21^ For this assay, THP‐1 cells were seeded at 0.4 × 10^5^ cells per well in 96‐well plates and differentiated for 72 h, and then loaded with cholesterol (10 μg mL^−1^) for 24 h as described in Section 2.3. Cholesterol‐loaded macrophages were treated with different concentrations of 6‐dihydroparadol (5, 10, 20, or 30 μm) or solvent vehicle (0.1% DMSO) for another 24 h. The cytotoxic natural product digitonin (50 μg mL^−1^) was used as a positive control and treatment was carried out for 4 h. Afterward, cells were incubated with PBS containing 10 μg mL^−1^ resazurin for 4 h. The relative cell viability was calculated from the increase in the fluorescence signal caused by the conversion product resorufin by measuring the fluorescence emission at 590 nm using an excitation wavelength of 535 nm with a Tecan GENiosPro plate reader.^21^

### 2.5 Protein Extraction and Western Blot Analysis

Protein extraction and Western blot analysis were performed, as described previously.^21^ THP‐1 cells were seeded at a density of 0.2 × 10^6^ mL^−1^ in a volume of 4 mL per well in 6‐well plates and differentiated for 72 h. Cells were washed twice with PBS, then loaded with cholesterol (10 μg mL^−1^) for 24 h. Cholesterol‐loaded macrophages were washed again with PBS and then treated with different concentrations of 6‐dihydroparadol (1, 5, 10, 20, or 30 μm), TO901317 (5 μm, positive control), or solvent vehicle (0.1% DMSO) for another 24 h. After treatment, cells were lysed in ice‐cold NP‐40 lysis buffer (150 mm NaCl, 50 mm HEPES (pH 7.4), 1% NP‐40, 1% PMSF, 0.5% Na_3_VO_4_, 0.5% NaF) containing cOmplete protease inhibitor (1%, Roche) for 30 min before centrifugation (16 060 g for 20 min) to remove cellular debris. Concentration of total cellular protein was measured according to the Bradford method using Roti‐Quant (Roche).

Samples (20 μg total protein per sample) were loaded and separated via SDS‐PAGE, and transferred to a polyvinylidene fluoride membrane. After blocking for 1 h with 5% nonfat dry milk in TBS‐Tween, membranes were incubated with the following primary antibodies at 4 °C overnight: ABCA1 (1:500), ABCG1 (1:500), SR‐BI (1:500), or actin (1:5000). After being washed with TBS‐Tween, membranes were incubated with goat anti‐mouse secondary antibody, HRP conjugate (1:5000) or anti‐rabbit IgG, HRP‐linked secondary antibody (1:500) according to the manufacturer's instructions. Protein bands were visualized with the Fuji LAS 3000 CCD camera (Fujifilm) and quantified with Multi Gauge software (Fujifilm).

### 2.6 RNA Extraction and Reverse Transcription‐Quantitative Polymerase Chain Reaction

THP‐1 cells were seeded at a density of 0.2 × 10^6^ mL^−1^ in a volume of 4 mL per well in 6‐well plates and differentiated for 72 h, then loaded with cholesterol (10 μg mL^−1^) for 24 h, as described previously.^21^ Cholesterol‐loaded macrophages were washed with PBS and then treated with different concentrations of 6‐dihydroparadol (5, 10 μm), TO901317 (5 μm, positive control), or solvent vehicle (0.1% DMSO) for another 24 h. After treatment, total RNA was extracted from cells using the peqGOLD Total RNA Kit. Concentration of total RNA was measured with NanoDrop 2000c. cDNA was synthesized from 1 μg total RNA based on the protocol from the High Capacity cDNA Reverse Transcription Kit. In combination with the LightCycler 480 System from Roche, LightCycler 480 SYBR Green I Master was used for quantification of ABCA1 and ABCG1 mRNA expression. Relative ABCA1 and ABCG1 mRNA levels were quantified with the ΔC_T_ method, using human 18S as an endogenous control.

### 2.7 Nuclear Receptor Luciferase Reporter Gene Transactivation

Transactivation experiments were performed in HEK293 cells. Cells (6 × 10^6^) were seeded in Φ15 cm dishes, cultured for 19 h, and then transfected by the calcium phosphate precipitation method^23^ with 6 μg of full‐length human LXRα/β, RXRα, or PPARγ expression plasmid, 6 μg of the respective firefly luciferase reporter construct (ABCA1‐Luc for LXRα/β, RXRE‐Luc for RXRα, or PPRE‐Luc for PPARγ), and 3 μg of the EGFP expression plasmid as internal control. After 6 h, the transfected cells were harvested and re‐seeded in 96‐well plates (5 × 10^4^ cells per well) in DMEM medium containing 2 mm glutamine, 100 U mL^−1^ benzylpenicillin, 100 μg mL^−1^ streptomycin, and 5% charcoal‐stripped FBS. Re‐seeded cells were treated with 6‐dihydroparadol (5 μm), positive control (GW3965 [1 μm], pioglitazone [5 μm], or bexarotene [1 μm]) or solvent vehicle (0.1% DMSO) for 18 h. The medium was then discarded and the cells were lysed with a reporter lysis buffer. The luciferase‐derived luminescence and the EGFP‐derived fluorescence were quantified with a Tecan Infinite M200Pro plate reader. The ratio of luminescence units to fluorescence units was calculated to account for differences in cell number or transfection efficiency. Results were expressed as fold induction compared to the solvent vehicle (0.1% DMSO) treatment group.

### 2.8 Quantification of ABCA1 and ABCG1 Protein Stability

THP‐1 cells were seeded at 0.2 × 10^6^ cells per well in a volume of 4 mL per well in 6‐well plates and differentiated as described in Section 2.7.^21^ Then, cells were loaded with unlabeled cholesterol as described above, and treated with 6‐dihydroparadol (10 μm) for 24 h. Control cells were treated with solvent vehicle (0.1% DMSO) for 24 h. Cells were lysed at different time points (0, 1, 2, 3, 4, and 6 h) after treatment with the protein synthesis inhibitor CHX (100 μm). The protein levels of ABCA1 and ABCG1 were detected by Western blot analysis. GraphPad Prism (version 4.03, GraphPad Software) was used to calculate the protein half‐lives (t_1/2_) based on a one‐phase exponential decay model.^24^

### 2.9 Degradative Pathway of ABCA1 Protein

THP‐1 cells were seeded at 0.2 × 10^6^ cells per well in a volume of 4 mL per well in 6‐well plates and differentiated as described in Section 2.8.^21^ Then, cells were loaded with unlabeled cholesterol as described above, and treated with 6‐dihydroparadol (30 μm) for 24 h and incubated for another 3 h with or without the proteasome inhibitor lactacystin at 10 μm, the lysosomal inhibitor chloroquine at 100 μm, or the calpain inhibitor calpeptin at 30 μg mL^−1^. Cells were lysed and the protein levels of ABCA1 were detected by Western blot analysis.

### 2.10 Statistical Analysis

For determination of differences between two groups, a two‐tailed unpaired Student's *t*‐test was applied after data were tested for normality. For multiple comparisons, data were analyzed by one‐way ANOVA followed by Bonferroni's post hoc test to compare means between groups. Two‐way ANOVA followed by Bonferroni's post test was used to analyze the effect of two independent variables. *p* < 0.05 was considered statistically significant. GraphPad Prism (version 4.03) was used for statistical analysis and figure generation.

## 3 Results

### 3.1 6‐Dihydroparadol Enhances Cholesterol Efflux from THP‐1‐Derived Macrophages Without Affecting Cell Viability

**Figure 2:**
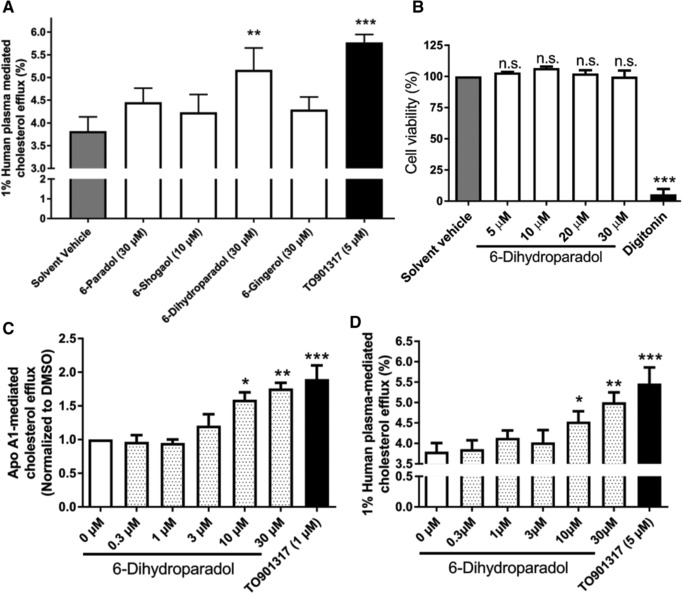
A) 6‐Dihydroparadol from ginger increases macrophage cholesterol efflux. THP‐1 cells were differentiated for 72 h with 200 nmol L^−1^ phorbol‐12‐myristate‐13‐acetate (PMA) and then loaded with cholesterol and radioactive cholesterol tracer ([^3^H]‐cholesterol) for 24 h. Cells were treated with 6‐paradol (30 μm), 6‐shogaol (10 μm), 6‐dihydroparadol (30 μm), 6‐gingerol (30 μm), TO901317 (5 μm, positive control), or the solvent vehicle (0.1% dimethyl sulfoxide [DMSO]) for another 24 h, then incubated with fresh serum‐free medium containing human plasma (1%, v/v) for 6 h, and cholesterol efflux was determined. B) 6‐Dihydroparadol does not affect cell viability of THP‐1‐derived macrophages. THP‐1 cells were differentiated as described in (A), and then loaded with unlabeled cholesterol for 24 h. Cells were treated with increasing concentrations of 6‐dihydroparadol (5–30 μm) for another 24 h. The viability was assessed by the resazurin reduction assay. Solvent vehicle treatment (0.1% DMSO) was used as a negative control. As a positive control, the cytotoxic natural product digitonin (50 μg mL^−1^, 4 h) was used. C,D) 6‐Dihydroparadol enhances both (C) apolipoprotein (apo) A1‐/ and (D) human plasma–mediated cholesterol efflux concentration dependently. THP‐1 cells were differentiated and loaded as described in (A). Cells were treated with increasing concentrations of 6‐dihydroparadol (0–30 μm) for 24 h. As a positive control, TO901317 (1 μm or 5 μm, 24 h) was used. Then macrophages were incubated with fresh serum‐free medium containing apo A1 (10 μg mL^−1^) or human plasma (1%, v/v) for 6 h, and cholesterol efflux was determined. The bar graphs represent mean ± SD from three independent experiments. *p < 0.05, **p < 0.01, and ***p < 0.001 versus control. n.s., not significant versus negative control (determined by Student's *t*‐test or ANOVA with Bonferroni's post hoc test).

We tested the pungent ginger constituents 6‐paradol, 6‐shogaol, 6‐dihydroparadol, and 6‐gingerol in cholesterol‐loaded THP‐1‐derived macrophages for their impact on cholesterol efflux. As shown in **Figure** 2A, only 6‐dihydroparadol (30 μm) enhances 1% human plasma–mediated cholesterol efflux (5.17%) significantly compared to the solvent vehicle (3.82%). 6‐Paradol, 6‐shogaol, and 6‐gingerol displayed a tendency to increase cholesterol efflux, which however did not reach significance. 6‐Shogaol at 20 μm and 30 μm shows obvious cytotoxicity against cholesterol‐loaded macrophages as observed under the microscope. TO901317 (5 μm, positive control), which is a well‐known LXR agonist, enhanced human plasma–mediated cholesterol efflux from cholesterol‐loaded macrophages strongly.

Next, we examined the influence of 6‐dihydroparadol on the viability of cholesterol‐loaded THP‐1‐derived macrophages by the resazurin conversion assay. Treatment with 6‐dihydroparadol from 5 to 30 μm for 24 h does not affect cell viability (Figure 2B). As expected, the cytotoxic natural product digitonin (positive control) clearly decreases cell viability at 50 μg mL^−1^ (Figure 2B).

We then assessed the effect of different concentrations of 6‐dihydroparadol (0.3–30 μm) on macrophage cholesterol efflux mediated by different acceptors (apo A1 and human plasma). As shown in Figure 2C,D, 6‐dihydroparadol promotes both apo A1‐ and human plasma–mediated cholesterol efflux from cholesterol‐loaded macrophages in a concentration‐dependent manner reaching significance at 10 μm.

**Figure 3:**
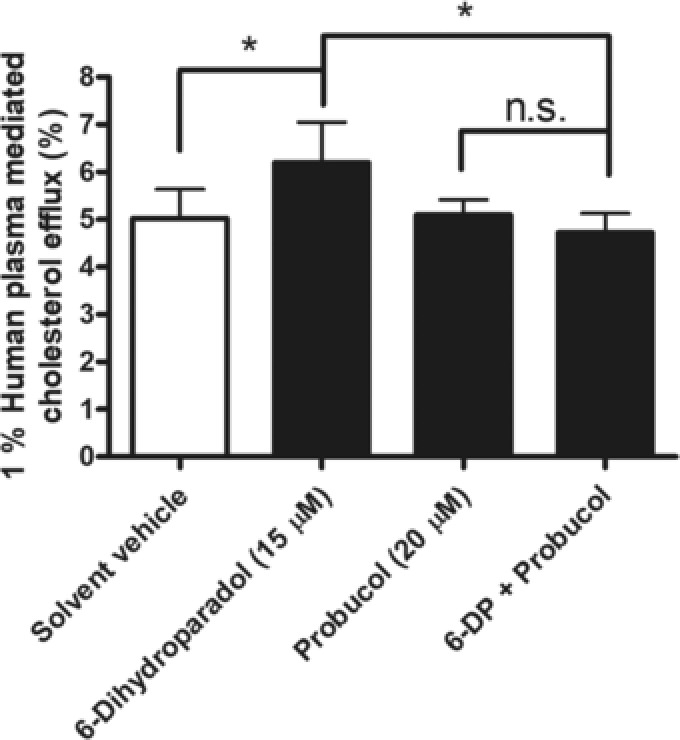
The ABCA1 inhibitor probucol completely abolishes 6‐dihydroparadol‐enhanced cholesterol efflux from THP‐1‐derived macrophages. THP‐1 cells were differentiated, loaded, and labeled as described in Figure 2. Cells were treated with 6‐dihydroparadol (6‐DP, 15 μm), probucol (20 μm), co‐treated with 6‐dihydroparadol and probucol or treated with the solvent vehicle (0.1% DMSO) for 24 h, then incubated with fresh serum‐free medium containing human plasma (1%, v/v) and corresponding compounds for another 6 h, and cholesterol efflux was determined. The bar graphs represent mean ± SD from four independent experiments. *p < 0.05 versus control. n.s., not significant versus control (determined by two‐way ANOVA with Bonferroni's post test).

We also evaluated the contribution of ABCA1 to the 6‐dihydroparadol‐increased cholesterol efflux by using the ABCA1 inhibitor probucol. As shown in **Figure** 3, probucol completely abolishes 6‐dihydroparadol‐enhanced cholesterol efflux from THP‐1‐derived macrophages.

### 3.2 6‐Dihydroparadol Increases ABCA1 and ABCG1 Protein Expression

**Figure 4:**
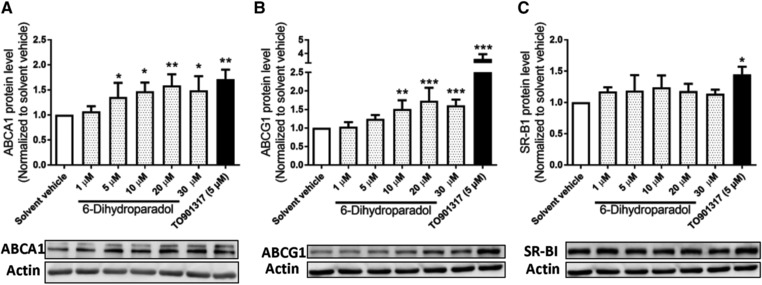
6‐Dihydroparadol increases A) ABCA1 and B) ABCG1, but not C) SR‐BI protein levels in cholesterol‐loaded THP‐1‐derived macrophages. THP‐1 cells were differentiated as described in Figure 2, and then loaded with unlabeled cholesterol for 24 h. Cells were treated with increasing concentrations of 6‐dihydroparadol (1–30 μm) for another 24 h. The protein levels of ABCA1, ABCG1, and SR‐B1 were detected by Western blot analysis. The control was treated with solvent vehicle (0.1% DMSO). As a positive control, TO901317 (5 μm, 24 h) was used. The bar graphs represent mean ± SD from three independent experiments. *p < 0.05, **p < 0.01, and ***p < 0.001 versus control (determined by Student's *t*‐test or ANOVA with Bonferroni's post hoc test).

The transporters ABCA1, ABCG1, and SR‐BI play very important roles in macrophage cholesterol efflux.^14^ We, therefore, tested the expression levels of these three transporter proteins in cholesterol‐loaded THP‐1‐derived macrophages treated with 6‐dihydroparadol. 6‐Dihydroparadol concentration‐dependently enhances both ABCA1 and ABCG1 protein levels significantly already at 5 μm (ABCA1) and 10 μm (ABCG1), respectively (**Figure** 4A,B). On the contrary, 6‐dihydroparadol does not affect SR‐BI protein levels at the tested concentrations (1–30 μm; Figure 4C). TO901317 (5 μm) increases ABCA1, ABCG1, and SR‐BI protein levels significantly compared to the solvent vehicle.

### 3.3 6‐Dihydroparadol Increases ABCA1 mRNA Levels, but Does Not Influence LXRα/β, RXRα, and PPARγ Activity

**Figure 5:**
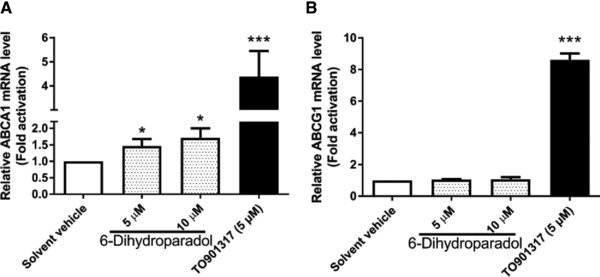
6‐Dihydroparadol increases A) ABCA1, but not B) ABCG1 mRNA levels in cholesterol‐loaded THP‐1‐derived macrophages. THP‐1 cells were differentiated as described in Figure 2, and then loaded with unlabeled cholesterol for further 24 h. Cells were treated with 6‐dihydroparadol (5 and 10 μm) for another 24 h. The control was treated with solvent vehicle (0.1% DMSO) for 24 h. As a positive control, the LXR agonist TO901317 (5 μm) was used. The mRNA levels of ABCA1 and ABCG1 were detected by RT‐qPCR. Bar graphs represent mean ± SD from three independent experiments. *p < 0.05 and ***p < 0.001 versus control (determined by Student's *t*‐test or ANOVA with Bonferroni's post hoc test).

**Figure 6:**
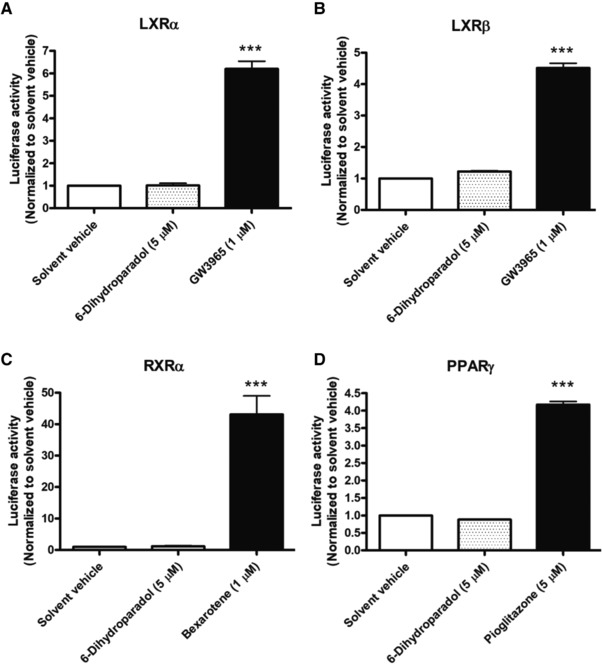
6‐Dihydroparadol does not influence A) LXRα, B) LXRβ, C) RXRα, and D) PPARγ activity. HEK‐293 cells were transiently co‐transfected with a plasmid encoding full‐length human LXRα/β, RXRα, or PPARγ, a reporter plasmid containing ABCA1‐Luc (for LXRα/β activity), RXRE‐Luc (for RXRα activity), or PPRE‐Luc (for PPARγ activity) and an EGFP expression plasmid as internal control. After 6 h, the transfected cells were harvested, re‐seeded, and treated with 6‐dihydroparadol (5 μm), positive control (GW3965 [1 μm], pioglitazone [5 μm], or bexarotene [1 μm]) or solvent vehicle (0.1% DMSO) for 18 h. The luciferase‐derived luminescence was normalized to the EGFP‐derived fluorescence, and the result is expressed as fold induction compared to the solvent vehicle. Bar graphs represent mean ± SD from three independent experiments each performed in quadruplicate. ***p < 0.001 versus control (determined by ANOVA with Bonferroni's post hoc test).

To determine whether the increased ABCA1 and ABCG1 protein levels correlate with respective mRNA levels, reverse transcription‐quantitative polymerase chain reaction (RT‐qPCR) experiments were performed. 6‐Dihydroparadol slightly but significantly increases ABCA1 mRNA levels at 5 μm and 10 μm (**Figure** 5A), while it does not influence ABCG1 mRNA levels at both concentrations in cholesterol‐loaded THP‐1‐derived macrophages compared to the solvent vehicle (Figure 5B). The positive control TO901317 (5 μm) increases both ABCA1 and ABCG1 mRNA levels significantly compared to the solvent vehicle. Thus, increased ABCA1 protein levels induced by 6‐dihydroparadol go in line with increased ABCA1 mRNA levels, while elevated ABCG1 protein levels are not mirrored by higher mRNA levels. *ABCA1* gene expression is positively regulated by the nuclear receptors LXRα/β, RXRα, and PPARγ.^15^ To examine whether increased ABCA1 mRNA level correlate with activation of these nuclear receptors, nuclear receptor luciferase reporter gene transactivation experiments were performed. 6‐Dihydroparadol (5 μm) does not influence LXRα, RXRα, and PPARγ activity (**Figure** 6A,C,D). It increased LXRβ activity by around 20% compared to the solvent vehicle. However, this difference did not reach statistical significance (Figure 6B). 6‐Dihydroparadol at 10 μm significantly decreased the number of transfected HEK293 cells.

### 3.4 6‐Dihydroparadol Increases the Half‐Lives of ABCA1 and ABCG1 Proteins

**Figure 7:**
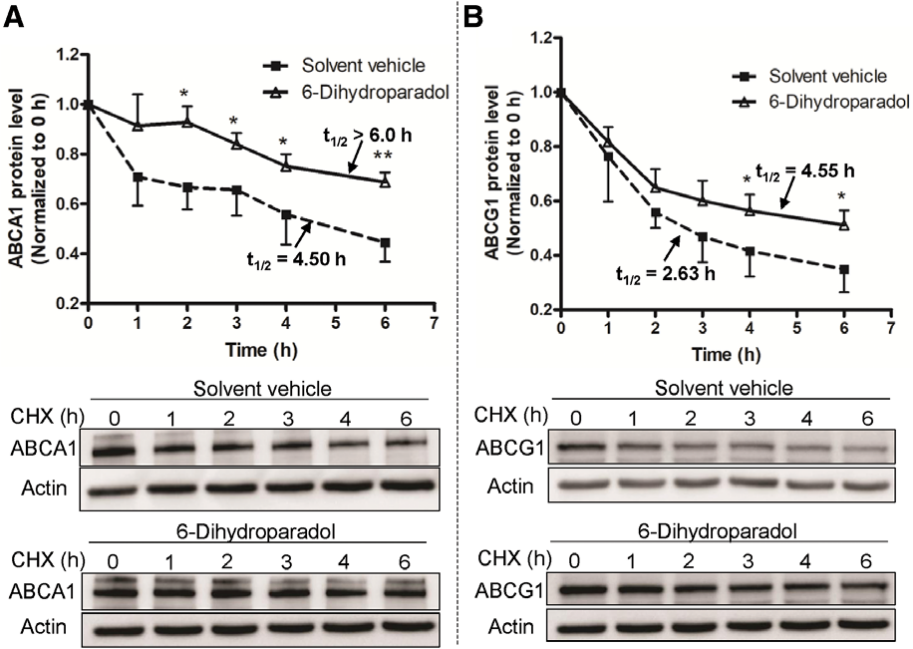
6‐Dihydroparadol increases A) ABCA1 and B) ABCG1 protein stability. THP‐1 cells were differentiated as described in Figure 2. Then cells were loaded with unlabeled cholesterol and treated with 6‐dihydroparadol (10 μm), or solvent vehicle (0.1% DMSO, control) for 24 h. Cells were lysed at different time points (0, 1, 2, 3, 4, and 6 h) after treatment with the protein synthesis inhibitor cycloheximide (CHX, 100 μm). The protein levels of both ABCA1 and ABCG1 were detected by Western blot analysis. The data points represent mean ± SD from three independent experiments. *p < 0.05 and **p < 0.01 versus control at the same time point (determined by Student's *t*‐test).

We next determined whether the observed changes in ABCA1 and ABCG1 protein levels induced by 6‐dihydroparadol are due to an increased protein stability in cholesterol‐loaded macrophages. Protein stability of ABCA1 and ABCG1 in the presence of 6‐dihydroparadol was tested by inhibiting de novo protein synthesis by CHX (100 μm) and monitoring the decay of the ABCA1 and ABCG1 protein over time (0, 1, 2, 3, 4, and 6 h). The t_1/2_ for ABCA1 protein in the presence and absence of 6‐dihydroparadol were determined to be 6.0 and 4.5 h, respectively (**Figure** 7A). The t_1/2_ for ABCG1 protein in the presence and absence of 6‐dihydroparadol were determined to be 4.55 and 2.63 h, respectively (Figure 7B). Thus, 6‐dihydroparadol significantly increases the half‐lives and thus protein stability of both ABCA1 and ABCG1 proteins in cholesterol‐loaded macrophages.

### 3.5 6‐Dihydroparadol‐Increased ABCA1 Protein Stability Might Result from an Impaired Proteasomal Protein Degradation System

**Figure 8:**
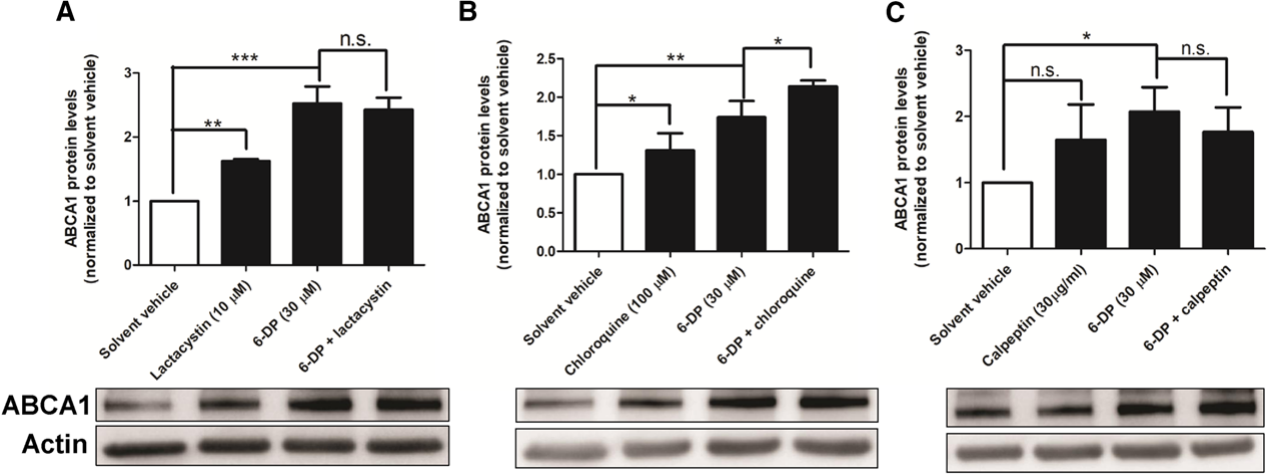
A–C) Effects of 6‐dihydroparadol and specific proteasomal, lysosomal, and calpain inhibitors on the ABCA1 protein levels. THP‐1 cells were differentiated as described in Figure 2, and then loaded with unlabeled cholesterol for 24 h. Cells were treated with or without 6‐dihydroparadol (6‐DP, 30 μm) for 24 h and incubated for another 3 h with or without the proteasome inhibitor lactacystin (10 μm), the lysosomal inhibitor chloroquine (100 μm), or the calpain inhibitor calpeptin (30 μg mL^−1^). The ABCA1 protein levels were detected by Western blot analysis. The bar graphs represent mean ± SD from three independent experiments. *p < 0.05, **p < 0.01, and ***p < 0.001 versus control; n.s., not significant versus control (determined by Student's *t*‐test or ANOVA with Bonferroni's post hoc test).

It was reported that the protein stability of ABCA1 is related to proteasomal, lysosomal, and calpain systems.^24^, ^25^, ^26^ We investigated the contribution of those pathways to 6‐dihydroparadol‐enhanced ABCA1 protein stability by the application of specific proteasomal, lysosomal, and calpain inhibitors. As shown in **Figure** 8, lactacystin (a proteasome inhibitor, 10 μm) and chloroquine (a lysosomal inhibitor, 100 μm) increase ABCA1 protein levels significantly. In addition, calpeptin (a calpain inhibitor, 30 μg mL^−1^) also tends to increase ABCA1 protein, however, without reaching statistical significance (Figure 8C). Addition of lactacystin could not further elevate the abundance of ABCA1 protein in cells treated with 6‐dihydroparadol (Figure 8A), suggesting that 6‐dihydroparadol might interfere with the proteasomal degradation pathway. In contrast, 6‐dihydroparadol had an additive effect on ABCA1 protein level upon co‐treatment with chloroquine, indicating that its mechanism of action is likely different from that of the lysosomal inhibitor chloroquine (Figure 8B). The data on calpain‐mediated degradation of ABCA1 suggested a minor, not significant contribution of this degradation pathway in our experimental setup. Thus, 6‐dihydroparadol‐increased ABCA1 protein stability might be associated with a reduced transporter degradation by the proteasomal system.

## 4 Discussion

The present study shows that 6‐dihydroparadol present in ginger enhances cholesterol efflux from THP‐1‐derived macrophages partly by stabilizing the transporter proteins ABCA1 and ABCG1.

Previous studies show that the ginger ethanolic extract significantly attenuated the development of atherosclerotic lesions in a model of apolipoprotein E‐deficient mice,^27^ and a model of rabbits on a high‐lipid diet.^28^ These studies suggest that protection from atherosclerosis by ginger correlates with a reduction of plasma cholesterol, triglyceride, VLDL and LDL levels, and with a decrease in the basal level of oxidized LDL, as well as with its susceptibility to oxidation and aggregation.^27^, ^28^ Subsequent studies indicated that ginger or constituents from ginger exhibited evidently hypolipidemic,^29^ anti‐inflammatory, and antioxidant activities.^2^ Our study now adds the finding that out of the four pungent compounds (6‐gingerol, 6‐shogaol, 6‐paradol, and 6‐dihydroparadol) from ginger, 6‐dihydroparadol enhances macrophage cholesterol efflux, which may contribute to the observed hypolipidemic potential of ginger. All four compounds share the same chemical skeletal structure. The difference between 6‐dihydroparadol and the other three compounds is a hydroxyl group at position 3, which appears pivotal for the positive effect of 6‐dihydroparadol on macrophage cholesterol efflux. Cholesterol efflux from macrophage foam cells is the first and important process in reverse cholesterol transport, whose increase may help to prevent or treat atherosclerosis.^30^ Our results suggest that dietary consumption of ginger rhizome might have a benefit in CVD, since the phenolic compound 6‐dihydroparadol present in ginger *rhizome* 31 enhances cholesterol efflux from cholesterol‐loaded macrophages. However, it needs to be noted here that in our in vitro studies, 6‐dihydroparadol starts to exhibit significant effects on macrophage cholesterol efflux only at concentrations >10 μm. Therefore, to reach effective concentrations of 6‐dihydroparadol systemically in vivo, the needed dosage of this compound would be quite high and most likely not achievable by dietary ginger.

Members of the ABC transporter family work in a synergistic manner to export cholesterol toward extracellular acceptors.^32^ ABCA1 mediates cholesterol efflux more efficiently to lipid‐poor apo A1, whereas ABCG1 and SR‐BI promote cholesterol export to mature HDL.^32^ Both ABCA1 and ABCG1 play major roles in mediating net cholesterol efflux from cholesterol‐enriched macrophages to HDL or serum.^33^ In contrast, cellular SR‐BI does not promote net cholesterol efflux from cholesterol‐loaded cells to plasma HDL.^34^ In addition, macrophages ABCA1 and ABCG1, but not SR‐BI, seem to promote macrophage reverse cholesterol transport in vivo.^35^ As shown here, 6‐dihydroparadol can increase ABCA1 and ABCG1, but not SR‐BI protein expression, suggesting that elevation of these two transporters is responsible for the 6‐dihydroparadol‐promoted cholesterol efflux from cholesterol‐enriched macrophages. Furthermore, we also found that the ABCA1 inhibitor probucol completely abolished 6‐dihydroparadol‐enhanced cholesterol efflux. Unfortunately, there are no studies directly showing that probucol does not influence ABCG1‐mediated efflux. In the study by Favari and coworkers,^22^ the authors observed no effect of probucol on cholesterol efflux in Fu5AH hepatoma cells that contain SR‐BI, but not functional ABCA1. Furthermore, they also showed that probucol inhibited cholesterol efflux from normal human skin fibroblasts but not from fibroblasts from a Tangier patient, who have defective ABCA1 transporters.^22^ These results suggest that the effect of probucol might be specific for ABCA1‐mediated cholesterol efflux.^22^ Another study used probucol for specific inhibition of the ABCA1‐mediated cholesterol efflux in wild‐type macrophages.^35^ However, due to lacking firm evidence that probucol does not influence ABCG1‐mediated efflux, it cannot completely be excluded that ABCG1 is also involved in the 6‐dihydroparadol‐induced cholesterol efflux. In addition, it is also possible that ABCG1 is involved in 6‐dihydroparadol‐increased cholesterol efflux in the absence of probucol, since the two ABC transporters act in a tandem manner.^32^

ABCA1 and ABCG1 protein levels in macrophages are highly regulated at both transcriptional and posttranscriptional levels.^36^ *ABCA1* and *ABCG1* gene expression can be regulated by the nuclear receptors LXR, RXR, and PPAR.^36^, ^37^, ^38^ Relevant posttranscriptional processes so far identified include modulation of ABCA1/G1 mRNA and protein stability.^39^, ^40^, ^41^ With respect to ABCA1, 6‐dihydroparadol increased both ABCA1 mRNA levels as well as ABCA1 protein stability, which is likely due to reduced proteasomal degradation. We also found that the observed increased ABCA1 mRNA levels were not due to nuclear receptor (LXRα/β, RXRα, and PPARγ) activation and thus likely due to activation of other transcription factors such as pregnane X receptor (PXR)^42^ or due to posttranscriptional effects such as altered mRNA stability. Increased ABCG1 protein levels in response to 6‐dihydroparadol were found not to be a result of altered mRNA levels, but of increased protein stability.

In summary, the present study examines for the first time the potential of pungent constituents from ginger on cholesterol efflux from cholesterol‐enriched human macrophages. Data show that 6‐dihydroparadol concentration‐dependently enhances cholesterol efflux from cholesterol‐loaded macrophages mediated by both apo A1 and human plasma. Furthermore, the 6‐dihydroparadol‐promoted efflux from cholesterol‐enriched macrophages might mainly be correlated with increased ABCA1 protein abundance due to elevated mRNA levels and enhanced protein stability. 6‐Dihydroparadol‐increased ABCA1 protein stability might be associated with decreased proteasomal degradation. This mechanism might contribute to the suggested antiatherogenic effects of the commonly used spice and flavoring agent, ginger.

## Conflict of interest

The authors declare no conflict of interest.
